# The impact of pulmonary artery to ascending aorta diameter ratio progression on the prognosis of NSCLC patients treated with immune checkpoint inhibitors

**DOI:** 10.3389/fimmu.2024.1302233

**Published:** 2024-01-29

**Authors:** Bingxin Gong, Yi Li, Yusheng Guo, Jing Wang, Weiwei Liu, Guofeng Zhou, Jiyu Song, Feng Pan, Lian Yang, Bo Liang

**Affiliations:** ^1^ Department of Radiology, Union Hospital, Tongji Medical College, Huazhong University of Science and Technology, Wuhan, China; ^2^ Hubei Key Laboratory of Molecular Imaging, Wuhan, China; ^3^ Department of Pathology, Union Hospital, Tongji Medical College, Huazhong University of Science and Technology, Wuhan, China

**Keywords:** pulmonary artery diameter, ascending aorta diameter, immune checkpoint inhibitors, non-small cell lung cancer, prognosis

## Abstract

**Background:**

Immunotherapy, represented by immune checkpoint inhibitors (ICIs), is a major breakthrough in cancer treatment. Studies have reported that the use of ICIs is associated with an increase in the pulmonary artery to ascending aorta diameter (PAD/AoD) ratio. However, the impact of PAD/AoD ratio progression on the prognosis of patients is unclear.

**Methods:**

This retrospective cohort study included patients with stage III or IV non-small cell lung cancer (NSCLC) treated with ICIs at the Wuhan Union Hospital between March 1, 2020, and September 1, 2022. The baseline and post-treatment PAD/AoD ratios of patients were evaluated through chest CT scans. The primary outcome of this study was overall survival (OS), while the secondary outcomes included progression-free survival (PFS), objective response rate (ORR) and disease control rate (DCR).

**Results:**

The PAD/AoD ratio increased after the initiation of ICIs (from 0.75 to 0.78; *P* < 0.001). A total of 441 patients were divided into severe group (n=221) and non-severe group (n=220) according to the median increase of PAD/AoD ratio (1.06). Compared with the non-severe group, the severe group had a lower DCR (87.8% vs. 96.0%, *P* = 0.005) and ORR (87.5% vs. 96.0%, *P* = 0.063). Over the entire duration of follow-up (median 22.0 months), 85 (38.5%) patients in the severe group and 30 (7.3%) patients in the non-severe group died. An increased PAD/AoD ratio was associated with shorter PFS (Hazard ratio (HR): 1.48 [95% CI, 1.14 to 1.93]; *P* = 0.003) and OS (HR: 3.50 [95% CI, 2.30 to 5.30]; *P* < 0.001). Similar results were obtained across subgroups.

**Conclusions:**

ICI treatment exacerbates an increase in the PAD/AoD ratio in patients with cancer, and greater increase in the PAD/AoD ratio was associated with a worse prognosis. PAD/AoD ratio could be a biomarker to stratify prognosis of NSCLC patients treated with ICIs.

## Introduction

Different from traditional anti-tumor drugs that directly attack cancer cells, immune checkpoint inhibitors (ICIs) enhance the tumor-killing response mediated by CD8-positive T cells through blocking the programmed cell death 1 (PD-1), programmed cell death-ligand 1 (PD-L1), or cytotoxic T-lymphocyte-associated protein 4 (CTLA-4) pathways (1, 2). Lung cancer is the most common malignant tumor, with more than 2 million cases and nearly 1.8 million deaths worldwide every year, of which approximately 85% of patients are diagnosed with non-small cell lung cancer (NSCLC) (3, 4). Since 2015, nivolumab became the first PD-1 inhibitor approved by the Food and Drug Administration (FDA) for the treatment of NSCLC and achieved good efficacy (5). The use of ICIs in patients with NSCLC has continued to expand (6, 7). However, despite its impressive efficacy, not all patients with NSCLC respond to ICIs, and excessive immune response leads to the destruction of immune tolerance and the occurrence of immune-related adverse events (irAEs) (8–10). These include pulmonary and cardiovascular adverse effects (11–13). Previously, we performed a retrospective analysis of 1,458 patients with NSCLC and found that patients treated with ICIs had a higher incidence of cardiovascular events than patients treated with other anti-neoplastic therapies, such as chemotherapy and targeted therapies. Recently, two cases have been reported in which pulmonary arterial hypertension (PAH) developed after ICI treatment of (14, 15). Therefore, there is an urgent need for a non-invasive biomarker that can predict the efficacy of ICI treatment to determine which patients will benefit from ICIs and avoid unnecessary irAEs.

As a measurable imaging marker that can be obtained in chest computed tomography (CT) images, the pulmonary artery to ascending aorta diameter (PAD/AoD) ratio is highly correlated with the pulmonary arterial pressure obtained by right heart catheterization, and its increase is a potential feature of PAH (16–18). It is also closely related to the change of cardiac structure and function (19–21). Recent studies found that the use of ICIs is associated with the enlargement of PAD and a consequent increase in the PAD/AoD ratio (22, 23). However, the association between an increased PAD/AoD ratio and the clinical outcomes remains unclear.

Therefore, our study aims to elucidate the relationship between the progression of PAD/AoD ratio and the prognosis of patients with NSCLC treated with ICIs, thereby clarifying whether the PAD/AoD ratio can be used as a biomarker to stratify prognosis of NSCLC patients treated with ICIs.

## Materials and methods

This retrospective cohort study was approved by the local ethics committee and the institutional review board of the Tongji Medical College of Huazhong University of Science and Technology (Institutional Review Board No. S054), and the study procedures were performed in compliance with Good Clinical Practice and the Declaration of Helsinki. The need for written informed consent was waived by the institutional review board.

### Study design and patient selection

This was a retrospective study conducted at a single academic center. Consecutive NSCLC patients treated with ICIs initially at Wuhan Union Hospital from March 1, 2020 to September 1, 2022 were included in this study. All patients underwent at least two CT scans (before and after treatment).

The inclusion criteria were as follows: (1) patients diagnosed with stage III or IV NSCLC following the NCCN Clinical Practice Guidelines in Oncology: Non-Small Cell Lung Cancer (24); (2) patients aged > 18 years; (3) patients had two or more chest CT scans; (3) patients received ICI treatment for more than 4 cycles. The exclusion criteria were as follows: (1) patients with incomplete or difficult-to-evaluate clinical or imaging data; (2) patients with other primary malignant tumors; (3) the time between two chest CT scans was less than three months.

### Procedures

We retrospectively collected baseline data of patients from clinical cases, including age, gender, body mass index, diabetes, hypertension, smoking, hyperlipidemia, hemoglobin, neutrophil to lymphocyte ratio, platelet to lymphocyte ratio, type of ICIs, and stages of tumor. All baseline information was based on the results of the first hospitalization. Data related to hypertension, diabetes and hyperlipidemia included those diagnosed during hospitalization and use of related medications.

### CT image acquisition

Imaging parameters were obtained from baseline and the most recent chest CT scan after initiation of ICIs. Two CT scans of all patients were acquired on a 128-slice CT scanner (Siemens Healthcare, Forchheim, Germany) using the same parameters. Patients were placed in a supine position and received sufficient breathing training before scanning. The scanning range was from the level of the upper thoracic inlet to below the costophrenic angle; the tube voltage was 120kVp and the tube current was automatically adjusted. All CT images were reconstructed using a standard soft convolution kernel with a slice thickness of 1 mm and an interval of 1 mm.

### PAD/AoD ratio measurement and assessment

**Figure 1:**
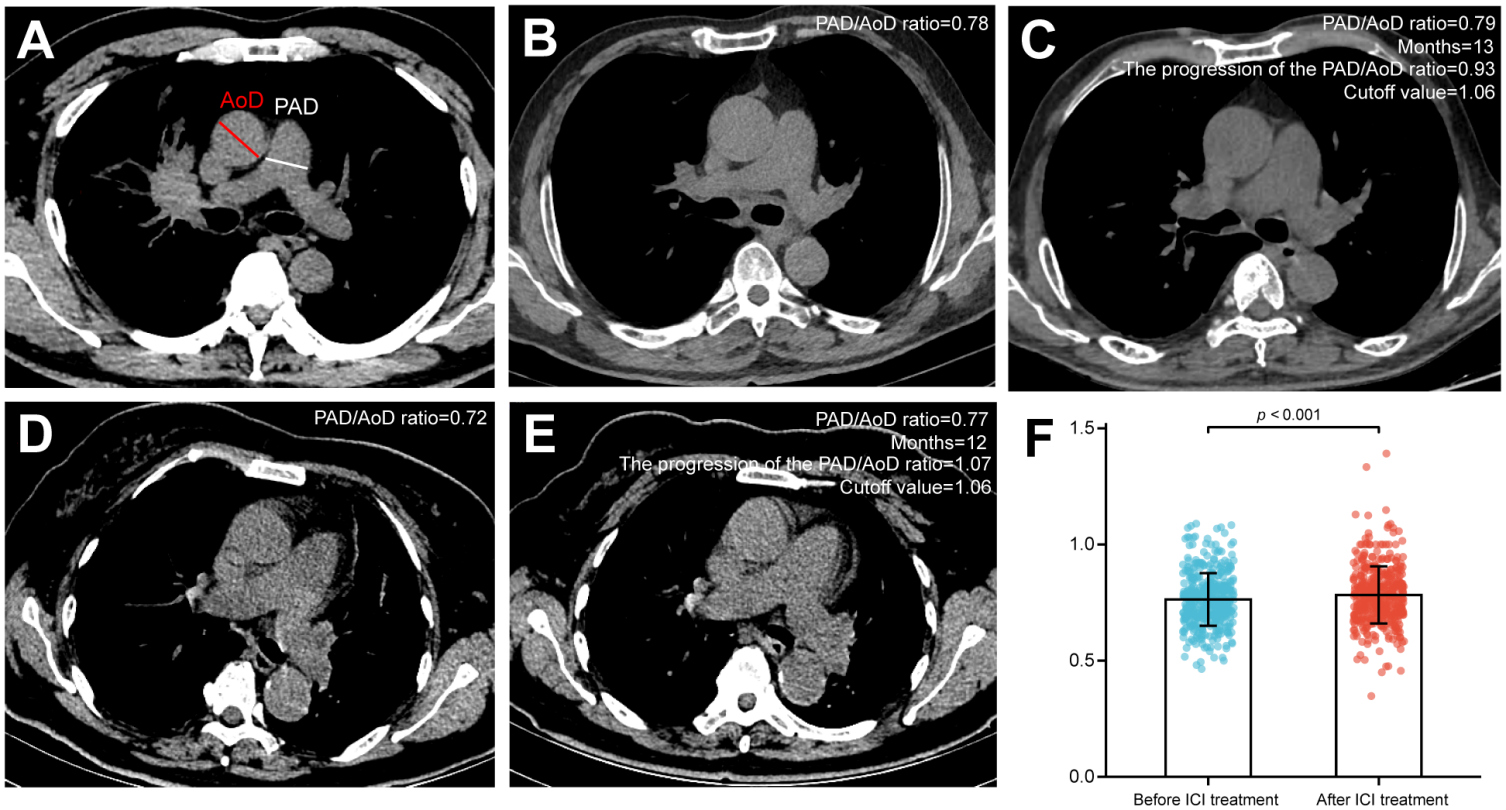
**(A)** Pulmonary artery diameter (PAD) and ascending aorta diameter (AoD) measured by chest CT scan. **(B, C)** The PAD/AoD ratio of a patient was 0.78 before immune checkpoint inhibitors (ICIs), which progressed to 0.79 after 13 months of ICI treatment. The progression of the PAD/AoD ratio was 0.93, which was less than the cutoff value (1.06), and this patient was included in the non-severe group. **(D, E)** The PAD/AoD ratio of a patient was 0.72 before ICIs, which progressed to 0.77 after 12 months of ICI treatment. The progression of the PAD/AoD ratio was 1.07, which was greater than the cutoff value (1.06), and this patient was included in the severe group. **(F)** Quantitation of the PAD/AoD ratio before and after ICIs. Data (n=441) were shown as means ± SD. Paired t-test was used to compare changes in the PAD/AoD ratio before and after ICIs.

According to the method described by Wells et al. (25), the inner diameter of the main pulmonary artery (PAD) and the ascending aorta (AoD) at the same level were measured (**Figure 1A**). Two independent radiologists (L.B. and Y.L. with 26 and 20 years of thoracic imaging experience, respectively) manually evaluated the images at the PACS workstation, blinded to all the clinical information. Assessments were repeated after 30 days, and intra- and inter-observer agreements were calculated. Any disagreements were resolved by discussion and consensus. The progression of the PAD/AoD ratio was annualized according to the following formula: [(post-ICIs/pre-ICIs)/months] *12. Using the 1.06 as the cutoff value, the patients were divided into severe group (PAD/AoD ratio ≥ 1.06) and non-severe group (PAD/AoD ratio < 1.06) (**Figures 1B–E**). The cut-off value of 1.06 was based on the median of PAD/AoD ratio increase. The median is a common and practical cut-off value classification method, which can be found in continuous variables such as clinical indicators (26), laboratory indicators (27, 28), and imaging indicators (29), and pathological indicators (30, 31). **Figure 1F** depicts the PAD/AoD ratio before and after ICIs with standard deviation and data points.

### Follow-up and end points

The required follow-up data was obtained through clinical cases information (including outpatient or inpatient information), imaging data (including CT and magnetic resonance (MR) images) and telephone follow-up. All patients were followed up until September 1, 2022 or until the patient death. The primary outcome of this study was overall survival (OS), defined as the time from ICI initiation to patient death from any cause. The secondary outcomes included progression-free survival (PFS), objective response rate (ORR) and disease control rate (DCR). The time from the patient starting ICI treatment to disease progression or death was expressed as PFS. Treatment efficacy was evaluated using complete response (CR), partial response (PR), stable disease (SD), and progressive disease (PD) criteria. The ORR and DCR were calculated from the proportion of patients with CR and PR and from those with CR, PR and SD, respectively. The above definitions are based on *New response evaluation criteria in solid tumours: Revised RECIST guideline (version 1.1)* (32).

### Statistical analysis

Continuous variables were presented as mean (SD) or median (IQR) and were compared using the t-test or the Wilcoxon rank sum test. Paired samples used paired t-test. Categorical variables were presented as counts (percent) and were compared using the Pearson χ2 test or the Fisher exact test. Baseline and post-treatment PAD/AoD ratios were compared by paired t-test. The log-rank test was used to compare the differences in PFS and OS between the severe and non-severe groups and the results were presented in the form of Kaplan-Meier survival curves. Univariable and multivariable Cox models were used to estimate hazard ratios for PFS and OS between the two groups. Variables identified as *P* < 0.1 in the univariable analysis were entered into the multivariable Cox regression model. Hazard ratios for PFS and OS were calculated for each subgroup using unstratified univariate Cox models with covariates of clinical interest. All statistical tests were two-tailed, with the *P* value level of significance being 0.05. The data were analyzed using SPSS software, version 26.0 (IBM, Chicago, IL, USA), and R software, version 4.3.0 (R Foundation).

## Results

### Patient characteristics

A total of 441 patients with NSCLC who received ICI treatment were included in this retrospective cohort study, including 221 patients with severe progression of PAD/AoD ratios and 220 patients with non-severe progression. The baseline demographic and clinical characteristics of the patients are shown in **Table 1**. The proportion of patients with a history of smoking in the severe group was higher than that in the non-severe group (30.8% vs. 23.8%, *P* = 0.004). With the exception of smoking history, there were no significant differences were observed in baseline characteristics between the two groups.

**Table 1:** Baseline characteristics of patients between the severe and non-severe groups.

Characteristics	Severe group	Non-severe group	*P* value
Patient characteristics			
Patients, n	221	220	
Gender, n (%)			0.304
Male	197 (44.7%)	189 (42.9%)	
Female	24 (5.4%)	31 (7%)	
Age, No. (%)			0.313
<65	115 (26.1%)	125 (28.3%)	
≥65	106 (24%)	95 (21.5%)	
Body mass index (kg/m2), n (%)			0.982
≤Median	180 (40.8%)	179 (40.6%)	
>Median	41 (9.3%)	41 (9.3%)	
PAD/AoD ratio, median (IQR)	0.75 (0.69, 0.81)	0.76 (0.69, 0.84)	0.291
Diabetes, n (%)	20 (4.5%)	22 (5%)	0.734
Hypertension, n (%)	68 (15.4%)	69 (15.6%)	0.893
Smoking, n (%)	136 (30.8%)	105 (23.8%)	0.004
Hyperlipidemia, n (%)	63 (14.3%)	72 (16.3%)	0.336
Hemoglobin (g/L), mean (SD)	122.9 (15.8)	125.8 (15.9)	0.055
Neutrophil to lymphocyte ratio, n (%)			0.240
≤2	32 (7.3%)	41 (9.3%)	
>2	189 (42.9%)	179 (40.6%)	
Platelet to lymphocyte ratio, n (%)			0.529
≤150	143 (32.4%)	136 (30.8%)	
>150	78 (17.7%)	84 (19%)	
Types of ICIs, n (%)			0.371
PD-1	206 (46.7%)	200 (45.4%)	
PD-L1	15 (3.4%)	20 (4.5%)	
Stages, n (%)			0.150
Stage III	71 (16.1%)	57 (12.9%)	
Stage IV	150 (34%)	163 (37%)	

PAD, pulmonary artery diameter; AoD, ascending aorta diameter; IQR, inter quartile range; SD, standard deviation; ICIs, immune checkpoint inhibitors; PD-1, programmed cell death protein 1; PD-L1, programmed cell death ligand 1.

### PAD/AoD ratio progression

During the entire follow-up period, the PAD/AoD ratio progressed from 0.75 (IQR, 0.69 to 0.82) before ICI treatment to 0.78 (0.71 to 0.85) after ICI treatment (*P* < 0.001, **Figure 1F**). In addition, we randomly selected 100 patients with NSCLC who receive non-ICI treatments at the same time and evaluated their PAD/AoD ratio before and after treatment to exclude the potential influence of factors other than ICI treatment. Baseline characteristics between ICI and non-ICI groups are summarized in **Supplementary Table 1**. There was no difference in baseline PAD/AoD ratio (0.75 vs. 0.76; *P* = 0.351) and the two CT scans intervals (12.0 months vs. 11.6 months; *P* = 0.279) between the two groups. The PAD/AoD ratio in the non-ICI group changed from 0.76 (IQR, 0.71 to 0.82) to 0.75 (IQR, 0.69 to 0.83) and was not statistically significant (*P* = 0.716; **Supplementary Table 2**). Intra-observer and inter-observer agreement for the PAD/AoD ratio measurement were both good at 0.96 (95% CI, 0.91 to 0.97) and 0.92 (95% CI, 0.87 to 0.95) respectively.

### Tumor response

Tumor responses between the two groups are shown in **Supplementary Table 3** and **Supplementary Figure 1**. The proportion of patients in the severe group with PR was 42.5%, with PD was12.2%, and with SD was 45.2%, compared with 51.4% (PR), 4.1% (PD), and 44.4% (SD) in the non-severe group. The DCR in the severe group was significantly lower compared to that in the non-severe group (87.8% vs. 96.0%, *P* = 0.005). At the same time, patients in the severe group also showed a tendency to have a lower ORR, although the difference was not statistically significant (87.5% vs. 96.0%, *P* = 0.063).

### Survival analysis

**Figure 2:**
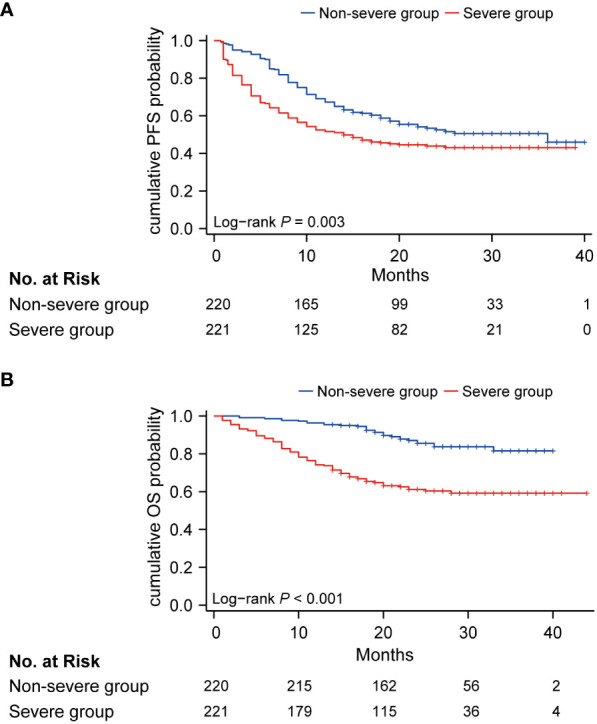
Kaplan-Meier curve of PFS **(A)** and OS **(B)** in non-severe group (blue) and severe group (red). PFS, progression-free survival; OS, overall survival.

During the median follow-up of 22.0 months (IQR 17.0, 29.0), 85 of 221 (38.5%) patients died in the severe group and 30 of 220 (7.3%) patients died in the non-severe group. Hazard ratios (HRs) for the severe group versus non-severe group were 1.48 (95% CI, 1.14 to 1.93; *P* = 0.003) for PFS and 3.50 (95% CI, 2.30 to 5.30; *P* < 0.001) for OS (**Figure 2**). Twenty-month PFS rates were 44.5% (95%CI, 38.4 to 51.6) versus 55.4% (95%CI, 49.1 to 62.6), and twenty-month OS rates were 63.2% (95%CI, 57.0 to 70.0) versus 89.7% (95%CI, 85.6 to 94.0). Median PFS was 14.0 (95%CI, 10.0 to not attained) and 36.0 (95%CI, 20.0 to not attained) months for the severe and non-severe groups, respectively. Due to insufficient follow-up time, the median OS of the two groups cannot be obtained. Instead, we plotted receiver operating characteristic (ROC) curves with OS at different time points and calculated the area under the curve (AUC) (**Supplementary Figure 2**). We constructed Kaplan-Meier curves for PFS and OS using the Youden index of 1-year, 1.5-year, and 2-year OS as the optimal cutoff value (**Supplementary Figure 3**), and obtained similar results as before.

### Univariable and multivariable analyses

In univariable analysis for PFS, severe PAD/AoD progression, diabetes, hemoglobin, neutrophil to lymphocyte ratio, platelet to lymphocyte ratio and stage were identified as potential predictors and further included in the multivariable model. In the multivariable Cox regression model, severe PAD/AoD progression (HR, 1.60 [95%Cl, 1.23 to 2.09]; *P* < 0.001) and higher disease stage (HR, 2.30 [95%Cl, 1.63 to 3.24]; *P* < 0.001) were significant risk factors for reduced PFS (**Table 2**). Similarly, the final multivariable Cox regression model for OS showed that severe PAD/AoD progression (HR, 3.84 [95%Cl, 2.52 to 5.83]; *P* < 0.001), advanced age (HR, 1.64 [95%Cl, 1.11 to 2.44]; *P* = 0.012), hypertension (HR, 1.51 [95%Cl, 1.02 to 2.23]; *P* < 0.039) and advanced disease stage (HR, 2.36 [95%Cl, 1.45 to 3.86]; *P* < 0.001) were independent risk factors associated with the reduced OS (**Table 3**).

**Table 2:** Univariate and multivariate Cox proportional hazards analyses for PFS.

Parameter	Univariate analysis		Multivariate analysis	
	Hazard ratio (95% CI)	*P* value	Hazard ratio (95% CI)	*P* value
Groups				
Non-severe group	Reference		Reference	
Severe group	1.48 (1.14,1.93)	0.003	1.60 (1.23, 2.09)	< 0.001
Gender				
Male	Reference			
Female	1.00 (0.67, 1.49)	0.993		
Age				
<65	Reference			
≥65	1.10 (0.84, 1.43)	0.493		
Body mass index (kg/m^2^)				
<Median	Reference			
≥Median	0.78 (0.54, 1.11)	0.165		
PAD/AoD ratio	0.94 (0.31, 2.84)	0.916		
Diabetes				
No	Reference		Reference	
Yes	1.44 (0.96, 2.16)	0.082	1.42 (0.94, 2.15)	0.092
Hypertension				
No	Reference			
Yes	1.19 (0.90, 1.56)	0.220		
Smoking				
No	Reference			
Yes	0.87 (0.67, 1.13)	0.289		
Hyperlipidemia				
No	Reference			
Yes	0.89 (0.67, 1.19)	0.429		
Hemoglobin (g/L)	0.99 (0.98, 1.00)	0.013	0.99 (0.99, 1.00)	0.122
Neutrophil to lymphocyte ratio				
≤2	Reference		Reference	
>2	0.64 (0.43, 0.95)	0.027	0.94 (0.62, 1.44)	0.788
Platelet to lymphocyte ratio				
≤150	Reference		Reference	
>150	0.63 (0.47, 0.84)	0.001	0.79 (0.57, 1.07)	0.129
Types of ICIs				
PD-1	Reference			
PD-L1	1.21 (0.77, 1.92)	0.414		
Stages				
Stage III	Reference		Reference	
Stage IV	2.33 (1.66, 3.27)	< 0.001	2.30 (1.63, 3.24)	< 0.001

PFS, progression-free survival; CI, confidence interval; PAD, pulmonary artery diameter; AoD, ascending aorta diameter; ICIs, immune checkpoint inhibitors; PD-1, programmed cell death protein 1; PD-L1, programmed cell death ligand 1.

**Table 3:** Univariate and multivariate Cox proportional hazards analyses for OS.

Parameter	Univariate analysis		Multivariate analysis	
	Hazard ratio (95% CI)	*P* value	Hazard ratio (95% CI)	*P* value
Groups				
Non-severe group	Reference		Reference	
Severe group	3.50 (2.30, 5.30)	< 0.001	3.84 (2.52, 5.83)	< 0.001
Gender				
Male	Reference			
Female	1.41 (0.75, 2.63)	0.284		
Age				
<65	Reference		Reference	
≥65	1.67 (1.16, 2.44)	0.006	1.64 (1.11, 2.44)	0.012
Body mass index (kg/m^2^)				
<Median	Reference			
≥Median	0.92 (0.57, 1.49)	0.736		
Baseline PAD/AoD ratio	0.43 (0.09, 2.06)	0.291		
Diabetes				
No	Reference			
Yes	1.60 (0.93, 2.75)	0.090		
Hypertension				
No	Reference		Reference	
Yes	1.66 (1.14, 2.41)	0.008	1.51 (1.02, 2.23)	0.039
Smoking				
No	Reference			
Yes	1.09 (0.75, 1.58)	0.647		
Hyperlipidemia				
No	Reference			
Yes	0.96 (0.65, 1.43)	0.851		
Hemoglobin (g/L)	0.99 (0.98, 1.00)	0.124		
Neutrophil to lymphocyte ratio				
≤2	Reference		Reference	
>2	0.59 (0.33, 1.06)	0.076	0.95 (0.52, 1.76)	0.875
Platelet to lymphocyte ratio				
≤150	Reference		Reference	
>150	0.66 (0.44, 0.99)	0.045	0.73 (0.48, 1.12)	0.148
Types of ICIs				
PD-1	Reference			
PD-L1	0.99 (0.50, 1.96)	0.981		
Stages				
Stage III	Reference		Reference	
Stage IV	2.11 (1.31, 3.42)	0.002	2.36 (1.45, 3.86)	< 0.001

OS, Overall survival; CI, confidence interval; PAD, pulmonary artery diameter; AoD, ascending aorta diameter; ICIs, immune checkpoint inhibitors; PD-1, programmed cell death protein 1; PD-L1, programmed cell death ligand 1.

### Subgroup analysis

**Figure 3:**
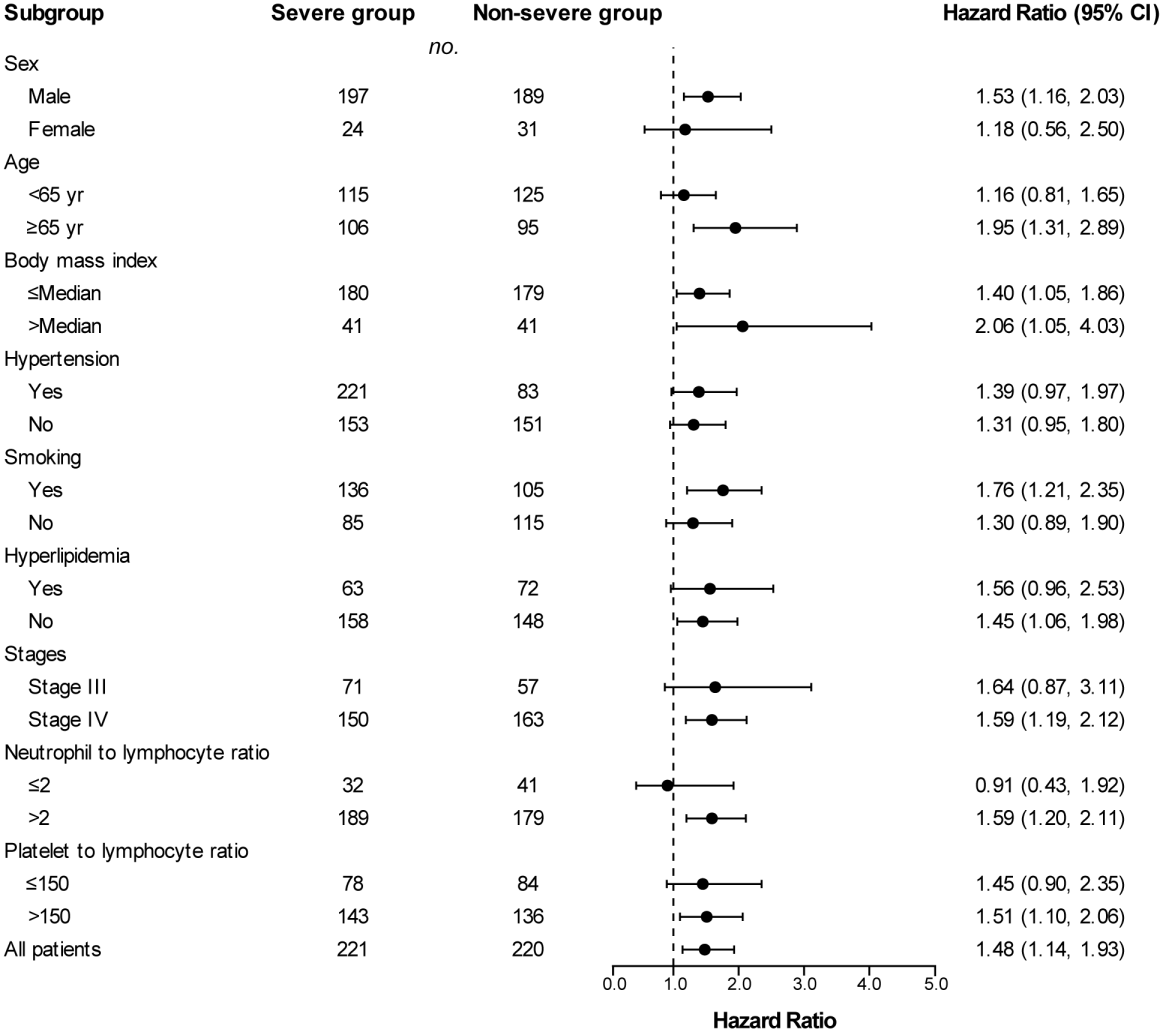
Subgroup analyses of progression-free survival between the severe and non-severe groups. Hazard ratios were derived from univariate cox model for each subgroup. Dashed line indicates Hazard ratio of 1.

**Figure 4:**
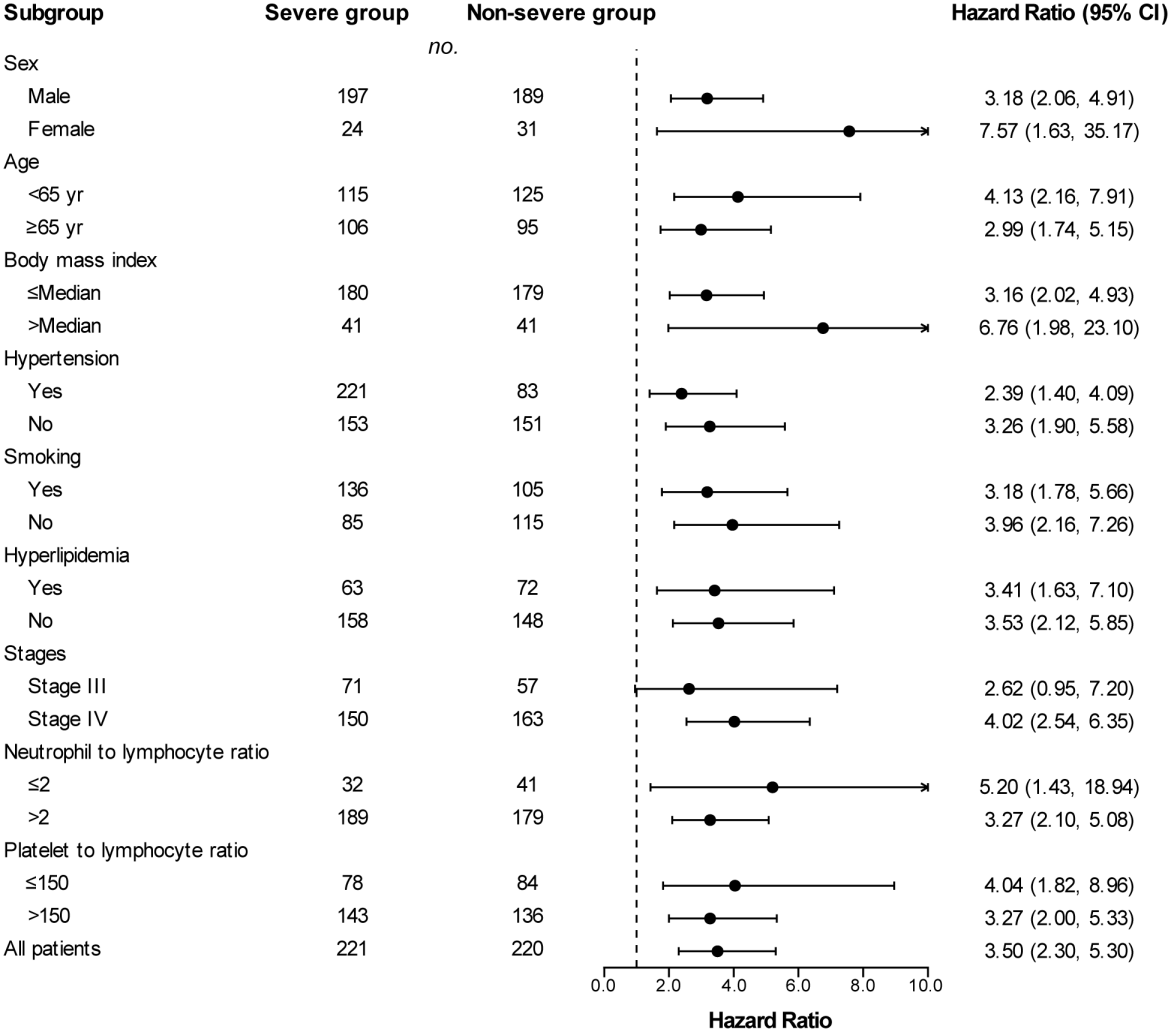
Subgroup analyses of overall survival between the severe and non-severe groups. Hazard ratios were derived from univariate Cox model for each subgroup. Dashed line indicates hazard ratio of 1.

Across subgroups based on baseline characteristics, a between-group difference in PFS (**Figure 3**) and OS (**Figure 4**) was consistently observed. In each subgroup, except for the subgroup of neutrophil to lymphocyte ratio ≤ 2, the severe group had a higher risk of shorter PFS than that in the non-severe group. However, in the OS analysis, the severe group presented a higher risk of shorter OS in all subgroups including the neutrophil to lymphocyte ratio ≤ 2 subgroup. Among patients with stage III disease, the severe group also had a tendency for higher risk of shorter PFS (HR, 1.64 [95%Cl, 0.87 to 3.11]) and OS (HR, 2.62 [95%Cl, 0.95 to 7.20]), although the difference was not statistically significant. PFS (HR, 2.06 vs. 1.40) and OS (HR, 6.76 vs. 3.16) in patients with obesity (body mass index ≥ Median) were more susceptible to PAD/AoD progression compared with patients without obesity (body mass index < Median), but the difference was not statistically significant.

## Discussion

In our study, the PAD/AoD ratio in patients with NSCLC before and after ICI treatment was compared, and the prognosis of these patients was followed up. Meanwhile, patients with NSCLC treated with other anti-tumor drugs during the same period served as standard controls and negative results were obtained, which is consistent with previous studies (23). The increase of PAD/AoD ratio accelerated after the initiation of ICIs. Compared with the non-severe group, the severe group had shorter OS and PFS, and the proportion of ORR and DCR was also lower. These results indicate that the PAD/AoD ratio could affect patient prognosis and can be used as a measurable and objective biomarker to predict the efficacy of ICI treatment. The underlying mechanism may be through the effects of ICIs on the cardiopulmonary system.

ICIs regulate CD8-positive T lymphocytes and regulatory T (Treg) cells by targeting the PD-1, PD-L1 and CTLA-4 pathways, thereby restoring the body’s tumor-suppressed immune function and enabling the body to produce an efficient immune response to tumor cells. However, immune activation caused by over-regulation of T cell activity breaks local or systemic immune homeostasis, produces autoinflammatory lesions, and leads to specific or non-specific damage to tissues and organs, thereby triggering autoimmune diseases and acute or chronic irAEs (33). Single-cell RNA sequencing and mass cytometry has shown that T cells are the predominant cell type in both human and mouse atherosclerotic lesions (34, 35). Meanwhile, compared to the blood counterparts, the differentiation, activation, and exhaustion of T cells in plaques were more pronounced. These “off-target” T cells activated by ICIs may promote the development of vulnerable atherosclerotic plaques through perforin- and granzyme B-mediated apoptosis of macrophages, endothelial cells, and smooth muscle cells (36), eventually leading to the occurrence of cardiovascular events. PAH is an autoimmune inflammatory disease to some extent. Although the role of T lymphocytes in the pathogenesis of PAH is controversial, the impact of cellular immunity on its occurrence and development cannot be ignored (37). Clinical and preclinical studies have shown that the production of antibodies that inhibit the PD-1, PD-L1, and CTLA-4 checkpoints activate T cells and lead to Treg cell dysfunction, ultimately leading to the development of PAH (37–40). Similarly, researchers found that there were a large number of activated and clonally expanded CD4+ and CD8+ T cells infiltrating the myocardium, cardiac conduction system and tumor tissue of patients with myocarditis caused by ICI treatment, suggesting that both cardiomyocytes and tumors express antigens can be recognized by monoclonal T cells (13, 41, 42). Activated T cells attack tumors, leading to autoimmune myocarditis and inflammation of the cardiovascular system. In addition, ICIs also cause damage to the cardiovascular system through mechanisms such as humoral immunity and cytokine induction (41). Cortellino et al. (43) used fasting mimicking diet (FMD) treatment to reverse cardiac fibrosis, necrosis and hypertrophy caused by ICIs, while reducing the immune infiltration of CD3+ and CD8+ cells in myocardial tissue.

This is the first study to report the relationship between PAD/AoD ratio progression and prognosis after ICI treatment. The PAD/AoD ratio increased 0.03 (from 0.75 to 0.78) after initiating ICIs. Similar to our results, Fournel et al. (23) found that the PAD/AoD ratio increased 0.05 (from 0.82 to 0.87) after the use of nivolumab. Although there were differences in baseline PAD/AoD ratio compared with our study, we believe that this was due to ethnic differences. This view can be confirmed in the study of Nguyen-Thu et al. (21). In their study, 193 Asian patients with clinical indications for the assessment of cardiovascular disease (CVD) or the cardiac function who underwent both coronary CT angiography (CCTA) and echocardiography had the baseline PAD/AoD ratio of 0.76 ( ± 0.12), which was similar to our study. In addition, Fournel et al. (23) found that two cases of life-threatening acute pulmonary hypertension occurred after ICI treatment, and their post-treatment PAD/AoD ratio were both >1. In our study, 19 patients had a PAD/AoD ratio >1 before ICIs, and this number increased to 27 after ICI treatment. Previous studies have demonstrated that PAD/AoD ≥1 was highly associated with pulmonary hypertension (44). PAD and AoD reflect pulmonary arterial pressure and heart function (21, 25, 45, 46). The expansion of the PAD/AoD ratio is a comprehensive manifestation of cardiovascular dysfunction, such as pulmonary vascular inflammation and decreased right heart function, which leads to increased pulmonary arterial pressure and luminal dilation and/or reduced aortic pressure and luminal constriction. Shibata et al. (19) found that the higher PAD/AoD ratio was associated with a lower left ventricular (LV) reverse remodeling (LVRR) (Odds ratio (OR): 0.948 [95% CI, 0.908 to 0.990]; *P* = 0.016), and the presence of LVRR represented a better prognosis. In the study of Nguyen-Thu et al. (21), higher PAD/AoD ratio was a potential risk factor for higher left ventricular mass (LVM) (Beta: 67.27 [SE, 30.534]; *P* = 0.03), left atrial volume (LAV) (Beta: 34.23 [SE, 10.23]; *P* < 0.001), and early mitral inflow velocity to mitral annular early diastolic velocity ratio (E/e’) (Beta: 5.08 [SE, 2.47]; *P* = 0.04), which were associated with worse cardiac function. Excessive activation of the immune system by ICIs can lead to immune cell infiltration of the myocardium and myocardial oxidative stress, resulting in reduced cardiac function. Reduced left ventricular function leads to a reduction in aortic diameter. Although right ventricular function may be reduced by ICIs, pulmonary function may also be affected (47), leading to an increase in right ventricular afterload and subsequent increases in both pulmonary artery pressure and pulmonary artery diameter. An association between ICIs and PAD/AoD ratio progression has been observed previously. The PAD and PAD/AoD ratio of 59 patients with advanced NSCLC increased after treatment with nivolumab (23). In another study, Mylvaganam et al. (22) conducted a retrospective analysis of 24 patients received ICIs including PD-1, PD-L1, and CTLA-4 antibodies. Their results showed significant increases in both right ventricular free wall longitudinal systolic strain (RVfwLS) and PAD/AoD ratio over a median treatment period of less than 100 days, suggesting that ICIs are associated with right ventricular dysfunction and vascular changes. However, their study did not provide survival data that is crucial for cancer research. Compared to these two studies, the number of patients in our study was > 7-fold and > 18-fold higher, respectively. In our study, a total of 441 participants (excluding standard controls) were included and followed up for 22 months (median follow-up) to obtain patient tumor response (ORR and DCR) and survival data (PFS and OS).

There were a few limitations to this study. This study was retrospective and conducted at a single center. Differences in patient admission and examination times may lead to selection bias. However, this study includes one of the largest numbers of ICI-treated patients reported to date. Furthermore, although we performed a complete survival analysis of patients, data pertaining to irAEs including pulmonary hypertension and cardiovascular events were not collected. In addition, we did not conduct separate analyzes on different ICIs, and there may be differences in PAD/AoD ratio progression among different ICIs. It has been reported that 50% of patients identified with immune-related PAH were treated with nivolumab (48). However, we randomly collected real-world patients with NSCLC treated with different ICIs, and the results are hence considered more generalizable. Finally, the changes in PAD/AoD ratio were generally low in this study, and there was a lack of hemodynamic parameter data obtained from invasive right heart catheterization, and cardiac function tests to corroborate our results. Therefore, more comprehensive prospective multicenter studies with longer follow-up periods are still needed to verify our results in the future.

## Conclusions

Our study found that PAD/AoD ratio progression accelerated after the initiation of ICIs, and greater increases were associated with worse clinical outcomes. The PAD/AoD ratio is expected to become an important indicator for evaluating the prognosis of patients treated with ICIs. Therefore, in clinical practice, we should routinely evaluate the PAD/AoD ratio in patients treated with ICIs to help early identify patients who could benefit from the treatment with ICIs versus those who could benefit from alternative treatment strategies and thereby guide clinicians in medication use.

## Data availability statement

The raw data supporting the conclusions of this article will be made available by the authors, without undue reservation.

## Ethics statement

The studies involving humans were approved by Tongji Medical College of Huazhong University of Science and Technology (Institutional Review Board No. S054). The studies were conducted in accordance with the local legislation and institutional requirements. The ethics committee/institutional review board waived the requirement of written informed consent for participation from the participants or the participants’ legal guardians/next of kin because this study is a retrospective study. Informed consent is not applicable in this study. The ethics committee approved the waiver of informed consent.

## Author contributions

BG: Writing – original draft, Writing – review & editing. YL: Writing – original draft, Writing – review & editing. YG: Writing – original draft, Writing – review & editing. JW: Writing – review & editing. WL: Writing – review & editing. GZ: Writing – review & editing. JS: Writing – review & editing. FP: Writing – review & editing. LY: Writing – review & editing. BL: Writing – review & editing.
