# Supplementary material for: The impact of pulmonary artery to ascending aorta diameter ratio progression on the prognosis of NSCLC patients treated with immune checkpoint inhibitors
Source: Front Immunol. 2024 Jan 29;15:1302233. doi: 10.3389/fimmu.2024.1302233 (PMC10859503; doi:10.3389/fimmu.2024.1302233)
Supplement: Supplementary file 1 [file DataSheet_1.docx]

**SUPPLEMENTAL MATERIAL**

Gong BX, Li Y, Guo YS et al. Prognostic value of pulmonary artery to ascending aorta diameter ratio in NSCLC patients treated with immune checkpoint inhibitors.

**Supplementary Table 1. Baseline characteristics between the patients with and without ICI treatment**

| Characteristics | ICI group | Non-ICI group | *P* value |
| --- | --- | --- | --- |
| Patient characteristics |  |  |  |
| Patients, n | 441 | 100 |  |
| Gender, n (%) |  |  | < 0.001 |
| Male | 386 (87.5%) | 72 (72.0%) |  |
| Female | 55 (12.5%) | 28 (28.0%) |  |
| Age, No. (%) |  |  | 0.917 |
| <65 | 240 (54.4%) | 55 (55.0%) |  |
| ≥65 | 201 (45.6%) | 45 (45.0%) |  |
| Body mass index (kg/m2), n (%) |  |  | 0.094 |
| ≤Median | 359 (81.4%) | 74 (74.0%) |  |
| >Median | 82 (18.6%) | 26 (26.0%) |  |
| PAD/AoD ratio, median (IQR) | 0.75 (0.69, 0.82) | 0.76 (0.71, 0.82) | 0.351 |
| Diabetes, n (%) | 42 (9.5%) | 13 (13.0%) | 0.299 |
| Hypertension, n (%) | 137 (31.1%) | 43 (43.0%) | 0.022 |
| Smoking, n (%) | 241 (54.6%) | 47 (47.0%) | 0.166 |
| Hyperlipidemia, n (%) | 135 (30.6%) | 34 (34.0%) | 0.509 |
| Hemoglobin (g/L), mean (SD) | 126 (114, 136) | 126 (113, 135) | 0.918 |
| Neutrophil to lymphocyte ratio, n (%) |  |  | 0.704 |
| ≤2 | 73 (16.6%) | 15 (15.0%) |  |
| >2 | 368 (83.4%) | 85 (85.0%) |  |
| Platelet to lymphocyte ratio, n (%) |  |  | 0.813 |
| ≤150 | 162 (36.7%) | 38 (38.0%) |  |
| >150 | 279 (63.3%) | 62 (62.0%) |  |
| Types of ICIs, n (%) |  |  |  |
| PD-1 | 406 (92.1%) |  |  |
| PD-L1 | 35 (7.9%) |  |  |
| Stages, n (%) |  |  | 0.420 |
| Stage III | 128 (29.0%) | 25 (25.0%) |  |
| Stage IV | 313 (71.0%) | 75 (75.0%) |  |

Abbreviations: PAD, pulmonary artery diameter; AoD, ascending aorta diameter; IQR, inter quartile range; SD, standard deviation; ICI, immune checkpoint inhibitor; PD-1, programmed cell death protein 1; PD-L1, programmed cell death ligand 1.

**Supplementary Table 2. PAD/AoD ratio progression between the patients with and without ICI treatment**

| Group | Baseline PAD/AoD ratio (IQR) | Post-treatment PAD/AoD ratio (IQR) | *P* value |
| --- | --- | --- | --- |
| ICI group | 0.75 (0.69, 0.82) | 0.78 (0.71, 0.85) | 0.009 |
| Non-ICI group | 0.76 (0.71, 0.82) | 0.75 (0.69, 0.83) | 0.716 |

Abbreviations: PAD, pulmonary artery diameter; AoD, ascending aorta diameter; IQR, inter quartile range; ICI, immune checkpoint inhibitor.

**Supplementary Table 3. Tumor response between the severe and non-severe groups**

| Tumor response | Severe group (n=221) | Non-severe group (n=220) |
| --- | --- | --- |
| CR, n | 0 | 0 |
| PR, n | 94 | 113 |
| SD, n | 100 | 98 |
| PD, n | 27 | 9 |
| DCR (%) | 87.5% | 96.0% |
| ORR (%) | 42.5% | 51.3% |

Abbreviations: CR, complete response; PR, partial response; SD, stable disease; PD, progressive disease; DCR, disease control rate; ORR, objective response rate.


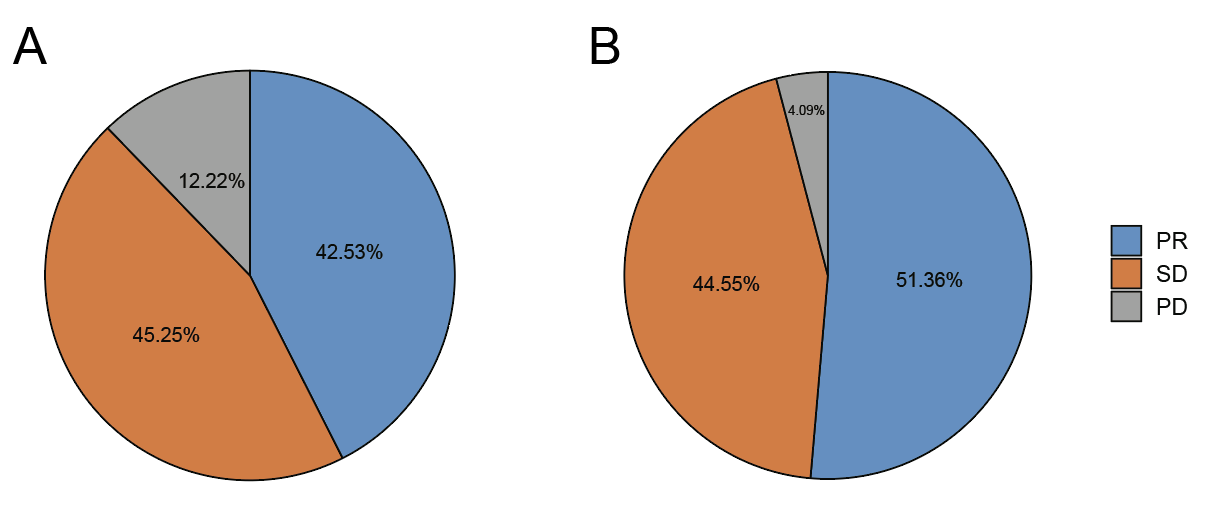


**Supplementary Figure 1** The pie chart illustrates percentage of partial response (PR), stable disease (SD), and progressive disease (PD) between the severe **(A)** and non-severe groups **(B)**.


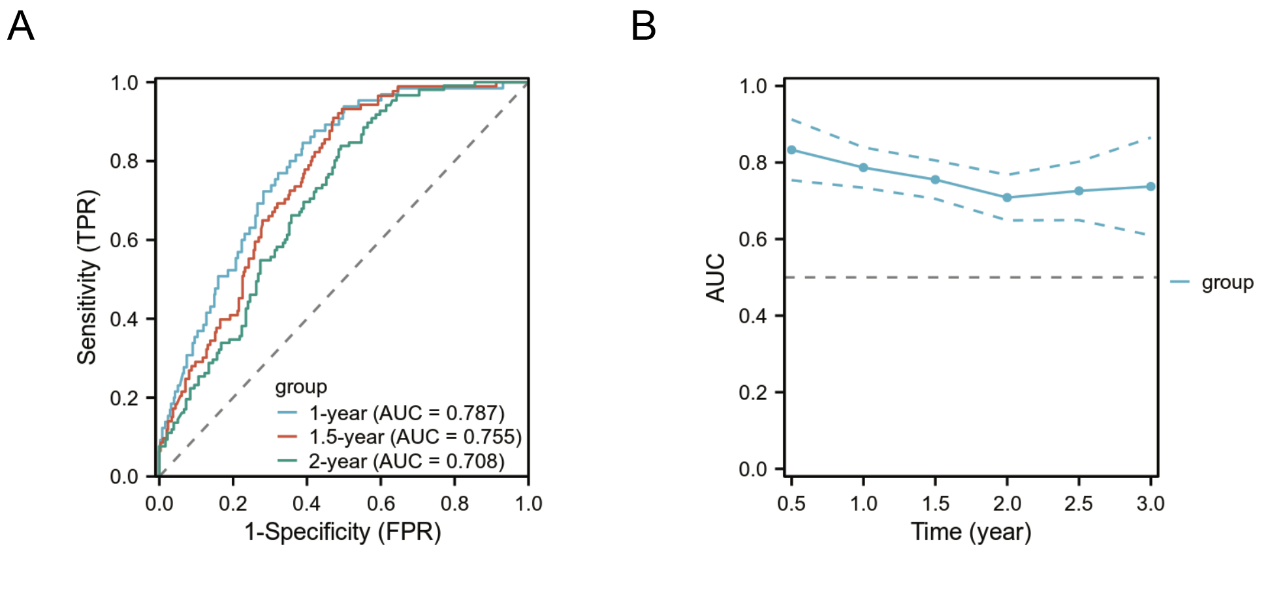


**Supplementary Figure 2** **(A)** Time-dependant ROC curves for OS at 1 year, 1.5 years and 2 years. **(B)** The AUCs of ROC curves for OS at different time points. ROC, receiver operating characteristic; OS, overall survival; AUC, area under the curve; TPR, true positive rate; FPR, false positive rate.


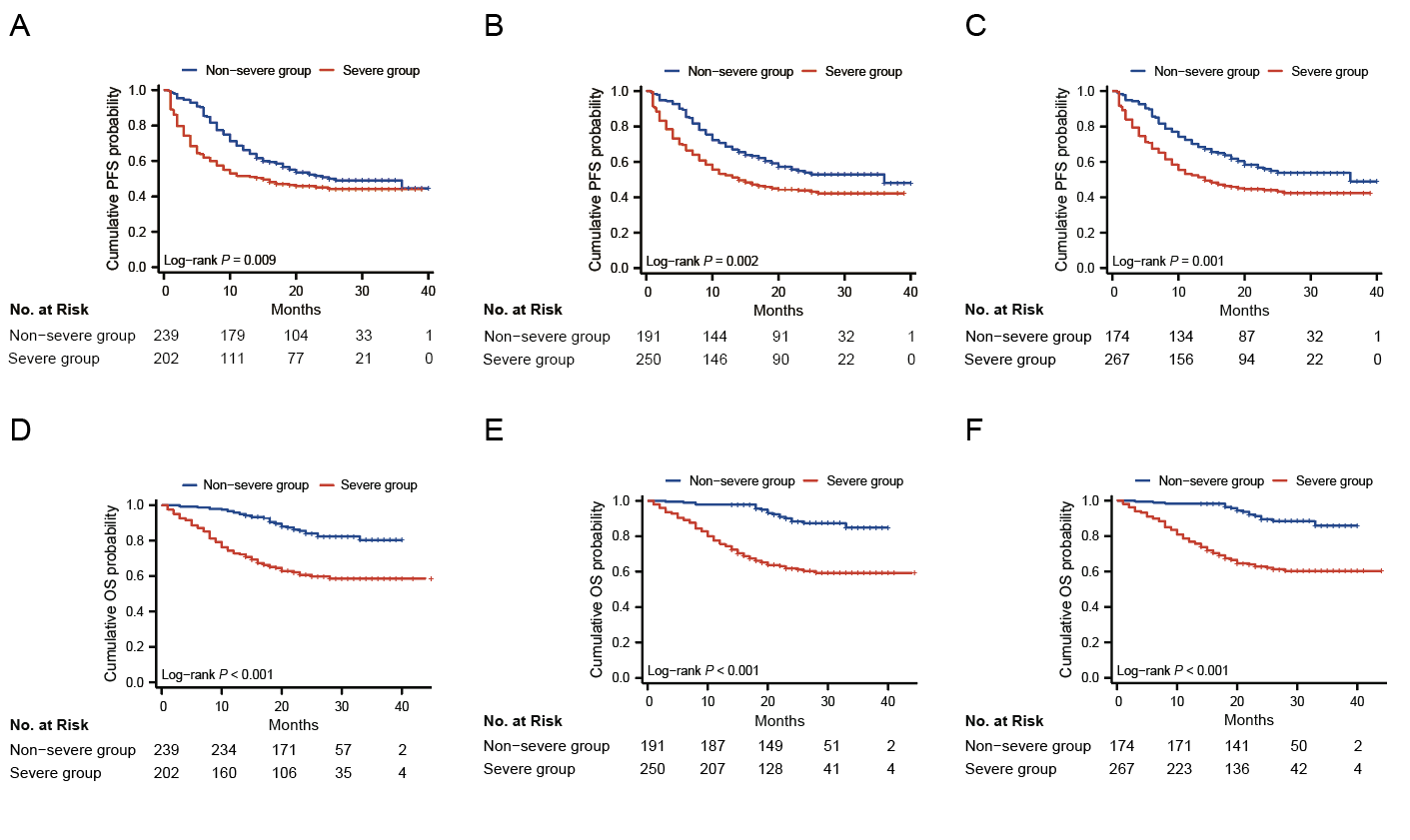


**Supplementary Figure 3** Kaplan-Meier curves of PFS and OS in non-severe group (blue) and severe group (red) were obtained using the Youden index of 1 year **(A, D)**, 1.5 years **(B, E)** and 2 years **(C, F)** OS as the optimal cutoff value. PFS, progression-free survival; OS, overall survival.
